# Gambling Behavior of Excluded Gamblers in a Multi-venue Exclusion System: Results from a Three-Wave Survey Conducted in Switzerland

**DOI:** 10.1007/s10899-023-10249-4

**Published:** 2023-09-19

**Authors:** Suzanne Lischer, Jürg Schwarz, Hannes Wallimann, Jacqueline Mathys

**Affiliations:** 1 School of Social Work, Lucerne University of Applied Sciences and Arts, Werftestrasse 1, Lucerne, CH-6002 Switzerland; 2 School of Business, Lucerne University of Applied Sciences and Arts, Lucerne, Switzerland; 3 Zurich, Switzerland

**Keywords:** Gambling disorder, Multi-operator-exclusion-program, Harm reduction, Prevention

## Abstract

Gamblers enrolled in the Swiss Multi-Venue Exclusion Program completed a written questionnaire three times, at six-month intervals. In addition to sociodemographic information, they provided details of their gambling behavior, and completed the South Oaks Gambling Screen-Revised (SOGS-R). The excluded gamblers were compared to a control group of non-excluded gamblers who also completed the questionnaire. The baseline survey demonstrated that there was a significant association between gamblers status (excluded *n* = 87 and non-excluded *n* = 259) and income (*p* = .039), as well as debt situation (*p* < .001) and SOGS-R score classification (*p* < .001). Over the course of three surveys, 242 gamblers participated. Of these, 133 respondents were not excluded from casinos at any time, 33 were excluded at the time of the first survey wave and remained so, while the exclusion status of 76 respondents changed over time, thus they were excluded for a minimum of one wave. Overall, 12.1% of excluded individuals stopped gambling altogether. Although exclusion is circumvented by some gamblers, it is associated with significant reductions in frequency, duration, and expenditure, as well as severity of problem gambling. The effects were more significant among gamblers who were excluded from casinos during the entire survey period. The results suggest that the duration of an exclusion should be at least six months instead of the current three months. 62.6% of the excluded gamblers had at least one exclusion lifted during the survey period. Further research is needed to investigate the implications of repeated exclusions for gambling-specific problems.

## Introduction

Exclusion programs are a host responsibility feature provided by gambling venues. Specifically, these programs aim to exclude gamblers at risk of, or experiencing, gambling-related harm from the respective gambling premises, for a specified period (Bellringer et al., 2010). They typically provide a self-exclusion option for individuals who perceive a loss of control over their gambling behavior and/or imposed exclusion in cases where problem gambling behavior or high losses are perceived by third parties, such as gambling operators or significant others (Kotter et al., 2017). Although the basic principles of game exclusion are consistent, programs vary significantly between countries because of different gambling environments and jurisdictional and legislative frameworks (Gainsbury, 2014; Parke et al., 2014). In many jurisdictions, exclusion programs are processed at the venue level, however, programs are beginning to transition to centrally administered systems, enabling simultaneous exclusion from multiple venues, especially in European jurisdictions (Håkansson & Åkesson, 2022; Pickering et al., 2018).

Self-exclusion is designed to prevent an individual from accessing gambling venues. As a preventive measure, it can help at-risk or problem gamblers regain control of their behavior or assist their efforts to abstain from at least one specific form of gambling for a certain time (Hayer & Meyer, 2011). Accordingly, reviews on this subject find reductions in gambling and gambling-related harms associated with self-exclusion (Drawson et al., 2017; Gainsbury, 2014; Kotter et al., 2018; McKnight, 2021; Motka et al., 2018). Interestingly, there do not seem to be significant differences in the effects of imposed exclusion over self-exclusion, with regard to gambling behavior (Kotter et al., 2017; Lischer & Schwarz, 2018). However, evidence suggests that after the exclusion period, many gamblers return to gambling (I. M. Cohen et al., 2011). Moreover, various studies indicate that the exclusion agreement is sometimes breached (Drawson et al., 2017), for example by some gamblers, who are excluded from land-based gambling, switching to gambling venues located just over the border (Lischer & Schwarz, 2018) or participating in illegal gambling (Lischer & Gebhard, 2018). Moreover, it is impossible to avoid the fact that gamblers who are excluded from online gambling can turn to unlicensed online casinos (Håkansson & Widinghoff, 2020).

It is important to note that the proportion of excluded gamblers who stop gambling altogether, due to exclusion, should not be used as the sole measure of effectiveness for exclusion programs (Nowatzki & Williams, 2002). In addition to changing gambling behavior, the effectiveness of programs can also be assessed by observing the curtailing of gambling problems (Hing and Nuske 2012). The effects of self-exclusion on reducing symptoms of problem gambling over the course of time, have also been demonstrated within several studies (Hayer & Meyer, 2011; Hing et al., 2015; Ladouceur et al., 2000, 2007; McCormick et al., 2018; Nelson et al., 2010; Pickering et al., 2018; Townshend, 2007). Using the South Oaks Gambling Screen (Lesieur & Blume, 1987), Ladouceur and colleagues reported significant reductions in symptoms of pathological gambling between enrollment and the first follow-up period six months later (Ladouceur et al., 2007). The effect of exclusion on the reduction of gambling problems has been evidenced in several reviews (Drawson et al., 2017; Gainsbury, 2014; Kotter et al., 2018; Ladouceur et al., 2017; McKnight, 2021; Motka et al., 2018).

Altogether, awareness of self-exclusion is more limited for online gambling than for more traditional land-based gambling activities (Håkansson & Henzel, 2020; Motka et al., 2018). Moreover, the vast body of evidence on gambling exclusion refers to self-exclusion for the most part (Gainsbury, 2014; Motka et al., 2018). This is related to the fact that the possibility of imposed exclusion as a preventive measure is not available in many jurisdictions (Kotter et al., 2017). In addition, since multi-venue exclusion programs are a relatively new phenomena and therefore not yet widespread, there is little evidence of their effectiveness. An important limitation in the current body of research is the fact that very few of the above-mentioned studies include a control group. Any observed effects cannot, therefore, be conclusively attributed to the exclusion. One study that does not include this limitation was presented by Hing and colleagues, who examined whether improvements in gambling behavior were due to self-exclusion or were the result of recognizing problem gambling behavior and committing to change, regardless of the intervention used. A methodological limitation of the study, however, is the varying length of time since the respondents were banned from gambling (Hing et al., 2015). Finally, it should be noted that although previous research has examined the effectiveness of this intervention, there is a dearth of studies investigating the processes and procedures involved at the termination of a self-exclusion agreement (Pickering & Blaszczynski, 2022; Williams et al., 2012).

### Exclusion Programs in Switzerland

In Switzerland, gambling is regulated by the Federal Gambling Act. With a revision of the law, which entered into force in January 2019, licensed Swiss casinos were given the opportunity to expand their license and also offer casino games online. The offering of lotteries and sports betting, both online and land-based, is provided by two Swiss lottery companies. The Federal Gambling Act requires every casino to develop a clear prevention strategy. Exclusions are imposed if proof can be found or there is a strong suspicion that gamblers are maintaining excessive debts, placing bets that are disproportionate to their financial circumstances, or experiencing other disruptions due to their gambling behavior. Alternatively, gamblers can also ask for a voluntary self-exclusion. The exclusion applies throughout Switzerland, and the casino must disclose gamblers’ identities to all other Swiss casinos. A national identification system automatically verifies the gambler’s identity when entering the gambling venue or registering on the gambling website and denies entry or registration to individuals who are banned, or who are minors. It is a multi-venue exclusion program, i.e., a banned gambler is excluded from land-based casinos and licensed online-casinos as well as online lotteries and sports betting. Nevertheless, some types of games are not part of the exclusion system, such as electronic lottery machines or local poker tournaments, as well as land-based lotteries. In principle, an exclusion is valid for an unlimited period. After three months, however, the voluntary self-exclusion may be lifted. The revocation of the imposed exclusion can be requested if the reason for it no longer exists. Prior to lifting any exclusion, a gambler must prove within an affordability check that he or she has no debts and sufficient financial means to participate in gambling. Moreover, he or she must complete an assessment with a qualified treatment provider. A personalized follow-up by the person responsible for prevention (employee of the respective provider), after the exclusion has been lifted usually lasts two months (Lischer & Schwarz, 2018; Swiss Federal Assembly, 2018). At the end of 2021, the number of gambling bans valid throughout Switzerland was 79,917 (Swiss Federal Gambling Board, 2022).

### Research Needs and Study Aims

The following study investigates the effectiveness of exclusion in Switzerland’s combined land-based and online, multi-operator exclusion system. Within the framework of a three-wave survey, the present study examines how the gambling behavior and gambling-related problems of excluded gamblers evolve over time. A group of non-excluded gamblers is also included, to control for the effects of the exclusion. To the best of our knowledge, this is the first study to examine the influence of exclusion upon gambling behavior, using this research design.

## Methods

### Data Collection

The survey was conducted in 19 of the 21 casinos in Switzerland and took place from September 2019 to July 2022. The research was conducted in all three language regions of Switzerland (German, French and Italian). In the framework of a three-wave study, gamblers from the land-based and online sectors, who are banned from Swiss casinos completed an online-questionnaire on three occasions at intervals of six months. The first survey (hereafter also referred to as T1) was conducted after the exclusion was implemented. The respondents were recruited by flyers that were handed out by the casino staff to raise awareness of the project. For those gamblers where online gambling was the determining factor for exclusion, the flyer was attached in the e-mail correspondence. Gamblers who agreed to participate subscribed themselves to a website. They then received an e-mail containing a link that gave them access to the online survey. Six months and then twelve months later, they received e-mails for the second and third surveys, respectively. The responses of excluded gamblers were compared to a control group of non-excluded casino gamblers who completed the questionnaire at the same time intervals. Those gamblers were recruited randomly through flyers distributed by casino employees. By implementing filters in the questionnaire, the non-excluded gamblers were not presented with questions about the exclusion. For all respondents (experimental and control group), participation in the first and second survey was rewarded with a shopping voucher of 20 Swiss Francs and 50 Swiss Francs for the third survey. The survey was conducted with the Unipark solution. The data is stored on a protected server at the Lucerne University of Applied Sciences and Arts.

### Instruments

#### Sociodemographic Data

The longitudinal survey consisted of demographic questions related to gender, age, Swiss language region, the highest level of education attained, employment status, net income, and the history of over-indebtedness.

#### Gambling Behavior

To measure gambling behavior, the questionnaire contained questions on respondents’ use of the different types of gambling products available in Switzerland and abroad during the past six months. A total of 25 game-categories were surveyed, which were condensed to a total of 10 categories for statistical analysis. Gambling frequency was investigated using six categories (less than once a month, one to three times per month, one to two times per week, three to four times per week, five to six times per week, daily). Gambling duration included six categories (less than one hour, one to two hours, three to four hours, five to six hours, seven to eight hours, and more than eight hours). To capture gambling expenditure, the category system of the Swiss Health Survey was adopted (less than 10 Swiss Francs, between 10 and 99 Swiss Francs, between 100 and 299 Swiss Francs, between 300 and 499 Swiss Francs, between 500 and 999 Swiss Francs, between 1,000 and 2,499 Swiss Francs, between 2,500 and 9,999 Swiss Francs, 10,000 Swiss Francs or more) (Swiss Health Survey 2017).

### Exclusion

Participants reported whether their exclusion was linked to land-based or online gambling or online-lotteries respective sport betting was the determining factor and gambling, and whether the exclusion was voluntary or imposed. In addition, the exact date of exclusion was requested and whether the exclusion had been lifted in the meantime.

### Problem Gambling

The original South Oaks Gambling Screen (SOGS) is a 20-item instrument used to screen for pathological gambling (Lesieur & Blume, 1987). The South Oaks Gambling Screen Revised (SOGS-R) is scored by summing the number of items endorsed out of 20. A cut-off score of five or more indicates that the respondent is a probable pathological gambler, whereas a score between one to four to indicates some problems. The answers refer to the last six months (Lesieur & Blume, 1993). Authorized German, French and Italian versions were used for the survey (Cremer et al., 2001; Lejoyeux, 1999; Lesieur et al., 1991).

### Sample

The sample size required for the study was estimated using a power analysis. An ANCOVA with repeated measures over the time points T1 to T3, with a factor for the groups, further factors and a covariate were assumed as the bases for the statistical test used in the data analysis. A significance level of 5% and a power (1- β) of 80% were assumed, and a medium effect size in the population was assumed, i.e., *f* = 0.25 (Cohen, 1992). The calculation with G*Power (Faul et al., 2007) shows that 80 subjects were needed for each of the two groups (excluded and non-excluded gamblers) at time T3.

### Statistical Analyses

Statistical analyses included basic statistics (mean, median, standard deviation, etc.) and statistical tests (chi-square test, Fisher’s exact test). To model complex relationships repeated measures ANOVA were calculated. Bonferroni correction was used to account for multiple comparisons. When prerequisites were not met, for example, when the normal distribution assumption was violated or when subsamples were small, nonparametric equivalents were used. Statistical significance level was set at α = .05. Analyses were performed using the statistical software R.

### Compliance with Ethical Standards

The Swiss Ethical Authority decided that the project did not require formal ethical approval since it did not involve research on human diseases or the structure and function of the human organism (file number Req-2019-00060). The participants provided their written informed consent to participate in this study. The data management plan was approved by the Swiss National Science Foundation.

## Results

### Characteristics of Excluded and Non-Excluded Gamblers at Baseline

Below is a comparison of the characteristics of the excluded and non-excluded gamblers at the time of the first survey. The distribution of respondents from the respective language regions roughly represented the distribution of the different language groups within Switzerland. Regarding gender, the percentage of males was slightly higher among excluded gamblers. The age of excluded gamblers ranged from 18 to 66 years, with a mean of 33.7 years and a median of 30 years and was thus almost congruent with the average age of non-excluded gamblers, which was 33.8 years (median 31 years), with a range of 18 to 84 years. The exclusion was not related to age, education level, form of employment, or nationality. In contrast, a significant association was found between the gambler status (excluded vs. non-excluded) and income (*p* = .039), as well as debt situation (*p* < .001), with the latter ranging from 10 Swiss Francs to 150,000 Swiss Francs. Moreover, the percentage of people who were banned from gambling abroad was higher among those who were excluded in Switzerland. Finally, Table 1 shows that a significant association between the gambler status and classification of the SOGS-R score (*p* < .001). On the one hand, the proportion of excluded gamblers with a SOGS-R score of ≥ 5 was substantially higher at 48.3% than that found for non-excluded gamblers, at 10.8%. On the other hand, the proportion of excluded gamblers with a SOGS-R score of 0 (the “no problem” gamblers), was substantially lower at 8.0% than that of non-excluded gamblers at 45.9%.

**Table 1:** Comparison of characteristics of excluded and non-excluded gamblers at baseline (N = 346)

	Excluded (*n* = 87)	Non-excluded (*n* = 259)	*p* value	% Missing
Male sex			.040*	0.3
Yes	80.2% (69)	67.8% (175)		
Language			.009*	0.0
German	64.4% (56)	47.1% (122)		
French	29.9% (26)	37.5% (97)		
Italian	5.7% (5)	15.4% (40)		
Age			.918	1.1
18–25	24.1% (21)	27.1% (69)		
26–45	59.8% (52)	58.4% (149)		
46–65	14.9% (13)	12.9% (33)		
66 and above	1.1% (1)	1.6% (4)		
Education			.447	2.3
Compulsory schooling	14.1% (12)	8.7% (22)		
Apprenticeship	41.2% (35)	39.1% (99)		
Diploma/College	16.5% (14)	24.1% (61)		
University degree	17.6% (15)	18.6% (47)		
Other	10.6% (9)	9.5% (24)		
Net income (Swiss Francs, per month)			.039*	5.2
Less than 3,000	14.8% (12)	24.3% (60)		
3,001–7,000	55.6% (45)	37.7% (93)		
7,100–9,000	17.3% (14)	22.3% (55)		
More than 9,000	12.3% (10)	15.8% (39)		
Form of employment			.091	1.4
Active	82.6% (71)	78.0% (199)		
Retired	0% (0)	2.4% (6)		
Welfare	3.5% (3)	0.4% (1)		
Student	8.1% (7)	12.9% (33)		
Others	5.8% (5)	6.3% (16)		
Citizenship			.682	11.3
Switzerland	67.5% (52)	70.9% (163)		
Other	32.5% (25)	29.1% (67)		
Debts due to gambling			< .001*	6.9
Yes	21.5% (17)	3.7% (9)		
SOGS-R			< .001*	0.0
No problem (0)	8.0% (7)	45.9% (119)		
Some problems (1–4)	43.7% (38)	43.2% (112)		
Prob. path. gamb. ≥ 5	48.3% (42)	10.8% (28)		
Exclusion abroad			.065	1.1
Yes	10.5% (9)	4.3% (11)		

Note: At the time of the surveys, the Swiss Franc and the Euro had an almost equivalent exchange rate.

We conducted all tests with a chi-square test except if the conditions were not fulfilled. In this case, we used Fisher’s exact test

* *p* < .05

**Table 2:** Type of exclusion

	Land-based	Online
Imposed exclusion	10.6% (5)	30% (12)
Voluntary exclusion	89.4% (42)	70% (28)

As noted above, the determining factor for exclusion was examined (land-based vs. online gambling). The *p-*value of the Fisher’s exact was found to be .031. Thus, more imposed exclusions were issued in the online gambling sector (see Table 2).

### Analysis of Gambling Behavior Over Time

N = 242 respondents participated in the three surveys. Of these, 55% (*n =* 133) of respondents were not banned at any time, 13.6% (*n* = 33) were banned at the time of the first survey wave and remained so, while 31.4% (*n =* 76) were excluded for a minimum of one wave. This group is hereafter referred to as “short-term excluded”. Of those short-term excluded, 19 individuals had been excluded between the time of survey waves.

### Gambling Behavior

Table 3 presents the results of the repeated measures ANOVA of the self-reported gambling behavior, i.e., frequency and duration, for different game types.

**Table 3:** Frequency and duration of self-reported gambling behavior over time for the three groups (Mean)

Game Type		Excluded (*n* = 33)			Non-excluded (*n* = 133)			Short-term-excluded (*n* = 76)			*p-*values
		T1	T2	T3	T1	T2	T3	T1	T2	T3	
Land based casino	F	6.9	0.5	0.3	5.3	5.3	4.1	6.2	6.9	4.7	GT: < .001; SV: < .001; GT*SV: < .001
	D	3.7	0.2	0.2	3.3	3.2	2.5	3.8	3.7	3.2	GT: < .001; SV: < .001; GT*SV: < .001
Licensed online games	F	9.9	1.4	0.6	1.5	1.6	2.5	3.1	2.9	4.2	GT: < .001; SV: .009; GT*SV: < .001
	D	2.2	0.5	0.4	0.5	0.5	0.7	1.1	0.9	1.1	GT: < .001; SV: .007; GT*SV: < .001
Swiss Lotto / Sportsbetting online	F	2.5	0.3	1.1	1.0	1.1	1.0	0.5	0.4	0.5	GT: .108; SV: .399; GT*SV: .031
	D	0.7	0.1	0.2	0.3	0.3	0.3	0.2	0.2	0.2	GT: .215; SV: .482; GT*SV: < .001
Land-based Lotto/ Sportsbetting	F	5.3	4.2	4.2	2.1	1.4	1.3	1.2	1.1	1.1	GT: < .001; SV: .099; GT*SV: .790
	D	1.0	0.7	0.7	0.6	0.5	0.5	0.3	0.3	0.2	GT: < .001; SV: .042; GT*SV: .502
International online games	F	5.6	4.9	3.0	2.6	1.8	0.9	4.7	2.8	2.5	GT: < .001; SV: < .001; GT*SV: .619
	D	2.0	1.3	1.1	0.8	0.5	0.4	1.5	0.8	0.9	GT: < .001; SV: < .001; GT*SV: .234
Gambling abroad	F	0.9	0.5	0.1	0.4	0.2	0.2	0.6	0.1	0.1	
	D	1.3	0.5	0.1	0.6	0.3	0.3	1.0	0.4	0.4	
Landbased poker	F	0.6	0.6	0.6	0.4	0.3	0.3	0.2	0.2	0.3	
	D	1.6	1.0	0.7	0.9	0.5	0.6	0.6	0.5	0.5	
Backroom	F	0.0	0.0	0.0	0.1	0.1	0.0	0.1	0.0	0.0	
	D	0.1	0.0	0.0	0.1	0.1	0.0	0.1	0.1	0.0	
Others	F	2.2	0.9	1.7	0.1	0.2	0.5	0.2	0.0	0.2	
	D	0.4	0.2	0.5	0.1	0.1	0.2	0.1	0.0	0.0	

GT and SV denote gambler types and surveys, respectively. The gambler types are excluded gamblers, non-excluded gamblers, and the short-term-excluded gamblers. The surveys distinguish between the first survey time point (T1), the second (T2), and the third (T3). F stands for frequency per month and D for the duration of participation in hours in a particular game

GT describes the three gambler types; “excluded”, “non-excluded”, and the so-called “short-term excluded”. The *p*-value GT indicates whether a significant difference in gambling behavior was found between the three groups. We can see that the frequency and duration of gambling participation significantly differs between the three gambler types except for Swiss lotteries and sports betting online (*p =* .108 and *p* = .215). The difference in gambling behavior for the three gambler types across the three survey time points is presented as SV (Survey). Significant differences were also observed regarding the time of the survey, except for lotteries and sports betting (land-based as well as online). Of particular interest, however, is the interaction between gambler types (GT) and time of survey (SV). Table 3 shows that the effect depends on gambler type for land-based casinos (*p* < .001 for duration and frequency), licensed online games (*p* < .001 for duration and frequency), and online lotteries and sports betting (*p* = .031 and *p* < .001, respectively). Moreover, it is important to note that the tests were limited to game types that had a frequency of more than two game participations per month.

Subsequently, the gambling behavior was examined in more depth according to game type. At T1, 81.1% of the excluded gamblers reported gambling in land-based casinos. As expected, the gambling participation of excluded gamblers in land-based casinos decreased significantly. 66.7% of excluded gamblers reported participating in licensed online gambling at time point T1. A decrease in game participation among excluded gamblers can also be observed in licensed online gambling. 39.4% of the excluded gamblers stated that they had participated in online lotteries and sports betting at T1. When the interaction between the gambler type and the observation period was considered, a significant change was observed, which can be attributed to the excluded gamblers. Land-based lotteries, on the other hand, including electronic lotteries and sports betting are not part of the multi-venue exclusion program. 60.6% of the excluded gamblers reported gambling land-based lotteries at time point T1. No significant change in their gambling behavior was detected regarding the interaction. At T1, 54.5% of the excluded gamblers reported having participated in unlicensed online games. Regarding participation in unlicensed online games, there was no significant difference found either among the three gambler groups or at the different survey wave time points. However, a portion of the excluded gamblers had circumvented their exclusion via corresponding offers. Indeed, at time point T3, 30.3% of the excluded gamblers indicated that they had participated in unlicensed online games. Overall, 12.1% percent of excluded gamblers stopped gambling altogether.

**Table 4:** Average monthly Gambling Expenditure per gambler type (percent of gambler per category)

Expenditure (in Swiss Francs)	Excluded (*n* = 33)			Non-excluded (*n* = 133)			Short-term-excluded (*n* = 76)		
	T1	T2	T3	T1	T2	T3	T1	T2	T3
< 10	0.0	6.1	6.1	9.0	11.3	16.5	1.3	2.6	1.3
10–99	9.1	18.2	27.3	21.1	25.6	16.5	2.6	5.3	5.3
100–299	9.1	3.0	6.1	29.3	22.6	29.3	27.6	31.6	38.2
300–499	0.0	9.1	0.0	21.1	21.8	23.3	38.2	42.1	32.9
500–999	18.2	15.2	12.1	6.8	9.0	4.5	14.5	11.8	11.8
1,000–2,499	33.3	12.1	21.2	6.0	3.0	1.5	7.9	2.6	2.6
2,500–9,999	15.2	6.1	3.0	0.0	0.0	0.8	5.3	0.0	1.3
≥ 10,000	9.1	6.1	3.0	0.0	0.0	0.0	0.0	0.0	0.0
Missing	6.1	24.2	21.2	6.8	6.8	7.5	2.6	3.9	6.6

The gamblers were asked about their gambling expenditure in the last six months. It was remarkable that the proportion of excluded gamblers with minor spending on gambling increased for the lower spending categories (less than 10 Swiss Francs as well as 10–99 Swiss Francs per month), while the proportion of those gamblers who spent high amounts (500 Swiss Francs or more per month) decreased.

### Problem Gambling Severity

**Fig. 1:**
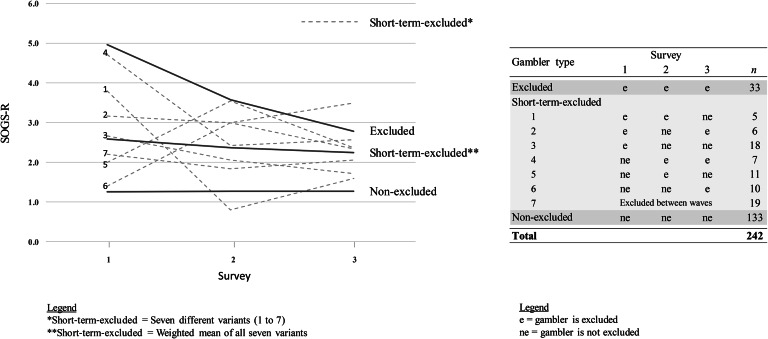
Evolution of SOGS-R scores of excluded, short-term-excluded and non-excluded gamblers over time

Figure 1 illustrates how the SOGS-R scores of the excluded and non-excluded gamblers evolved over time. Permanently excluded gamblers (*excluded*, *n* = 33) exhibited the highest levels of SOGS-R scores. Gamblers who had never been excluded (*non-excluded*, *n* = 133) showed the lowest SOGS-R score level; and gamblers who were excluded at least once (*short-term-excluded*) represented by the dashed lines 1 to 7, showed varying levels. A statistical analysis with repeated measures ANOVA revealed that the elapsed time (*Survey1*, *Survey2*, *Survey3*) in general had a significant influence on the level of the slope of the SOGS-R score, *F*(2, 478) = 3.615, *p* = .028. However, it is noted that for excluded gamblers, the decrease of the slope is greatest, the slope for non-excluded gamblers is constant and for those who were excluded at least once, the slope is fluctuating. Therefore, the effect of time significantly depends on the type of gambler, *F*(4, 478) = 6.085, *p* < .001. The type of gambler (*excluded, short-term excluded, non-excluded*) has a significant influence on the level of the slope of the SOGS-R score, *F*(2, 239) = 34, *p* < .001. The SOGS-R score level was highest for the excluded gamblers, and lowest for the non-excluded gamblers. The SOGS-R score levels for those who were excluded at least once were in between and showed changing trajectories. Moreover, the post hoc tests based on the Bonferroni correction revealed that all pairwise differences of the gambler types (*excluded, short-term excluded, non-excluded*) were significant to a value of *p*_adj_ <.001.

Among the excluded gamblers, the SOGS-R score decreased from a mean of 4.97 (median = 5, range 0–12) at time point T1 to 3.58 (median = 4, range 0–10) at T2 to 2.79 (median = 1, range 0–9) at the third survey time point (T3). A decrease in score values can also be observed among the short-term excluded respondents, but much less markedly. This group recorded a mean of 2.59 (range 0–11) at time point T1, a mean of 2.37 (range 0–8) at T2, and a mean of 2.25 (range 0–10) at T3. The median across all three measurement time points has been found to be 2. For the non-excluded gamblers, the mean at time point T1 is 1.26 and at both time points T2 and T3 it is 1.27. The median at the first time point is 0 and at subsequent measurement time points it is 1. The range of all three surveys is between 0 and 10.

## Discussion

The objective of this study was to evaluate the effectiveness of an exclusion in reducing gambling participation (frequency, duration, expenditure on gambling), using both an experimental and a control group, and differentiating gambling behavior by game type. A secondary objective was to assess the effect of exclusion upon the severity of gambling problems. Regarding the characteristics of the excluded gamblers, no significant discrepancies can be reported in comparison with other studies. In terms of gender, the percentage of males was slightly higher among excluded gamblers. The average age of the excluded gamblers was 33.7 years (median: 30 years). This is slightly lower than the age range found in other studies, which report an average exclusion age in the early or mid-forties (Kotter et al., 2018). 82.6% of the excluded gamblers were employed. This result is congruent with other studies. For instance, one review reports that the majority of self-excluders (73-90%) were either in full-time or part-time employment (Motka et al., 2018). 67.5% of the excluded respondents stated that they were of Swiss nationality. The actual proportion of Swiss citizens within the general population, however, may be somewhat lower (Lischer et al., 2014). In line with a recent review (Drawson et al., 2017), this study found that improvements related to a decrease in gambling frequency, duration and expenditure were all observed and maintained 12 months later, among the excluded gamblers. However, gambling despite exclusion was still relatively common. Due to entry controls, gambling in land-based casinos was almost non-existent among excluded gamblers. In the online sector, some gamblers bypass the registration controls that have been put in place for licensed online gambling. Overall, however, participation in licensed online games also fell significantly. With respect to online lottery and sports betting, game participation decreased less markedly, however, the interaction of gambler type and time of survey is still significant. The greatest levels of exclusion circumvention were seen in unlicensed online gambling. This observation has also been found in other studies (Håkansson & Widinghoff, 2020). 29.3% of the excluded gamblers continued to circumvent exclusion by gambling through unlicensed online providers. Interestingly, only a few excluded gamblers reported that they circumvented the ban in foreign casinos and gambling arcades. These results differ from previous studies, which found that some of the excluded gamblers continued to gamble abroad. The deviation may be due to methodological reasons: for example, in a previous study, transcripts of the exclusion lifting interviews were evaluated (Lischer & Schwarz, 2018). However, the extent to which gamblers circumvented their exclusions also becomes particularly apparent when one considers the monthly expenditures of the excluded individuals for gambling, which in some cases were still considerable in the third wave of the survey. For example, 21.2% of the excluded gamblers still reported gambling expenditures between 1,000 and 2,499 Swiss Francs in the third survey wave. Overall, 12.1% percent of excluded gamblers stopped gambling altogether. This result is lower than the value from a previous study, in which 20.5% of those excluded ceased all gambling activities (Kotter et al., 2017). However, from early on, it was pointed out that exclusion is an effective measure for individuals who have difficulty controlling their gambling behavior, and thus exclusion does not necessarily imply complete abstinence (Townshend, 2007).

According to the SOGS-R, 48.3% of the excluded respondents met the criteria for pathological gambling, 43,7% were considered at-risk gamblers and 8.0% reported having no gambling problems. This score is lower than that reported in previous studies, which have found that individuals who were self-excluded from land-based gambling were between 51% and 95% classified as persons experiencing gambling-related problems (Hayer & Mayer, 2011; Kotter 2017; Ladouceur 2000; 2007; Nelson 2010). It is likely that the lower value in this study is due to preventive measures such as early detection processes applied by land-based and online casinos, but also by lotteries. Across the three survey waves, the median of the SOGS-R score of the excluded gamblers decreased from 5 (T1), to 4 (T2) and finally to 1 (T3). The SOGS-R score of the so-called short-term excluded gamblers also decreased, but less markedly. These findings are consistent with a previous study, which found that those who were excluded for only six months reported a significantly smaller reduction in Problem Gambling Severity Index (PGSI) scores compared with those who were excluded for longer than six months (McCormick et al., 2018). In the context of the SOGS-R, the other striking aspects are as follows; that at the time of the first wave of the survey, 10.8% of non-excluded gamblers were classified as probable pathological gamblers. Overall, the mean scores across the three measurement points are 1.26 and 1.27, respectively, and the medians are 0 and 1 (range 0–10). Thus, there seem to be a few outliers in the group of non-excluded gamblers. Either the non-excluded gamblers with a high SOGS-R score had not yet been detected within the early detection process, or they had been able to prove by means of an affordability check that they did not have any debts and had sufficient financial means to participate in the game. Overall, it can be stated that excluded gamblers differed significantly from non-excluded gamblers in terms of problem gambling behavior. The preventive measures, and the early detection processes seem thus to be proving their worth.

Finally, it should be emphasized that in addition to the use of the preventive measure of exclusion, there are other factors that may have an influence on gambling behavior, such as the uptake of professional help. At the second measurement time point T2, a total of *n* = 23 individuals who had a SOGS-R score of $\ge$1, stated, to have sought help in the past six months. Of these, *n* = 14 individuals were excluded. A statistical analysis with repeated measures ANOVA revealed that among these gamblers, however, help-seeking had no significant effect on gambling behavior (duration, frequency, expenditure on gambling). A study with the same sample, however, found that exclusion has indeed a motivating effect on help-seeking (Lischer et al., 2023).

### Limitations

Several limitations that may have influenced the results should be identified. Firstly, there may have been a selection bias. For example, the proportion of gamblers with high incomes is likely to be disproportionate. Also, the required sample size of excluded gamblers could not be achieved due to limitations in recruitment. However, an analysis of the achieved effect sizes (Cohen’s *f*) shows that an effect worth reporting can also be obtained with a smaller sample size. Namely, the effect sizes vary between medium and large for the ANOVA with repeated measures of self-reported gambling behavior (frequency and duration) for the leading game types (*Casino land-based* and *License Online Games*). Furthermore, since there were gamblers who were only excluded for one or two waves or whose exclusion was revoked at least once, the gambling behavior of these participants was considered separately (see Fig. 1). The gambling behavior, as well as the SOGS-R score in this group varied, sometimes considerably. Given that the sample sizes of these individual variations (*n* = exclusions provoked per wave) were too small for a statistical analysis, the different variations were considered in a combined manner (short-term excluded). This procedure allowed a conclusive comparison between the gamblers who were excluded over all measurement time points with those who were excluded for a short term. Nevertheless, the results on the influence of short-term exclusions on gambling behavior must be interpreted with caution. In Addition, it is likely that the Covid pandemic influenced the results. During the survey period, land-based casinos were closed from March to June 2020 and from November 2020 to January 2021. This was taken into account by asking gamblers to consider only the time outside the lockdown in terms of game participation. Finally, as recruitment of respondents took place in the land-based and online casino environment, which may be one reason that individuals participating in online lotteries and sports betting were underrepresented in the sample.

## Conclusion

The game participation of excluded gamblers for the game types which are included in the multi-venue exclusion system decreased significantly, thus this regulation system is proving its worth. Admittedly, exclusion continues to be circumvented, but in broad terms, exclusion has been associated with reductions in frequency, duration, and gambling expenditures, as well as the severity of problem gambling. However, regulators should increase their efforts to block unlicensed online gambling operators from the market. As noted above, the Federal Gambling Act prescribes an indefinite period of exclusion, but a revocation of the voluntary exclusion can be requested after three months and, in the case of imposed exclusions, when the reason no longer exists. Although a reduction in gambling frequency, duration and problem gambling was also found among the short-term excluded gamblers, the effects were less significant than among gamblers who were excluded for the entire survey period. Thus, the question of the appropriate length of the exclusion deserves more in-depth consideration. The crucial decrease in the severity of gambling problems happened between the second and third measurement time points. Therefore, the results suggest that it takes at least six months for gambling behavior to stabilize enough to have an impact on reducing problem gambling behavior. This result is in line with previous research, which suggests that exclusion agreements should last at least six months to allow individuals sufficient time if they wish to seek treatment or to manage their gambling problems (Gainsbury, 2014). It can thus be concluded that the possibility of application for lifting the exclusion should be made only after six months. Therefore, consideration should be given to adjusting the duration of the exclusion within policy.

Notably, 62.6% of the excluded gamblers surveyed had at least one exclusion lifted during the survey period. It can thus be considered that there are obviously gamblers who try to regulate their gambling behavior by repeatedly excluding themselves. It is possible that some gamblers see self-exclusion as a way to temporarily control their difficulties, without desiring additional support. Some use this strategy repeatedly, for short periods of time (Tremblay et al., 2008). From a harm reduction perspective, the option of repeated exclusions should not be restricted. However, further research is needed to investigate the implications of repeated exclusions on gambling behavior and gambling-specific problems in greater detail.

## Data Availability

The dataset generated by the survey research and analyzed during the current study is available in the SWISSUbase repository, 10.48573/9fp4-fw62.
